# Peer Review of “Exercise-Induced Hypoalgesia Following Proprioceptive Neuromuscular Facilitation and Resistance Training Among Individuals With Shoulder Myofascial Pain: Randomized Controlled Trial”

**DOI:** 10.2196/45036

**Published:** 2022-12-27

**Authors:** Pattanasin Areeudomwong

**Affiliations:** 1 Department of Physical Therapy Mae Fah Luang University Chiang Rai Thailand

**Keywords:** exercise induced hypoalgesia, proprioceptive neuromuscular facilitation, PNF, resistance exercise, conditioned pain modulation, myofascial pain syndrome, resistance training, hypoalgesia, exercise-induced hypoalgesia, shoulder myofascial pain, myofascial pain, pain management, chronic pain, musculoskeletal pain, physical therapy, physiotherapy, shoulder pain, upper back pain, exercise, pain

*This is a peer-review report submitted for the paper “Exercise-Induced Hypoalgesia Following Proprioceptive Neuromuscular Facilitation and Resistance Training Among Individuals With Shoulder Myofascial Pain: Randomized Controlled Trial”*

## Round 1 Review

### General Comments

This paper [1] shows the effects of several types of exercise on exercise-induced hypoalgesia and conditioned pain modulation in patients with myofascial pain syndrome (MPS) of upper trapezius muscle. I thoroughly reviewed this work and find that there is room for improvement. The comments are as follows.

### Specific Comments

#### Major Comments

1. Why was this sample size chosen?

2. Regarding the first inclusion criteria, the patients must report their MPS for at least 4 weeks up to 3 months. In my opinion, the patients do not know whether they have MPS, and they commonly complain about their shoulder pain only. They will be informed about having MPS after being diagnosed by the physicians. Thus, it is not clear what the statement, “reported MPS” is referring to. In addition, I am not sure if patients with cardiovascular conditions, such as uncontrolled hypertension, are excluded from the study. Is it because of some risks of cardiovascular problems while performing accidental events during exercises such as holding the breath or due to resistant exercise–induced cardiovascular problems?

3. I am not sure how 60% maximum voluntary contraction (MVC) or pain-free load is set for each exercise intervention. Can we set it with other intensities? Is it possible that the patients doing an exercise with 60% MVC or pain-free load show significant differences on the outcomes? Why does the proprioceptive neuromuscular facilitation (PNF) method use 60% MVC for designing the PNF training protocol? The PNF intensity is set as a maximal resistance of the patients to facilitate an optimal outcome. Some techniques, such as the hold-relax technique with maximal resistance but within subpain threshold, are effectively used for relaxing muscle spasm or guarding in patients with muscle pain conditions. The authors apply agonist reversal, combination of isotonic contraction, and rhythmic stabilization without providing details of start and end positions, thus making it difficult to follow.

#### Minor Comments

##### Abstract

1. Please provide a specific name for a muscle affected by MPS, such as “patients with MPS of upper trapezius muscle.”

2. Exercise-induced hypoalgesia should be defined in relation to what outcomes are included for it.

3. Please specify specific area of pressure pain threshold of remote sites on the extensor carpus radialis and the peroneus longus.

4. Is the visual analog scale one of the study's outcomes?

## Abbreviations

MPS: myofascial pain syndrome
MVS: maximum voluntary contraction
PNF: proprioceptive neuromuscular facilitation
